# Comparison of clinical antibiotic susceptibility testing interpretations to CLSI standard interpretations

**DOI:** 10.1017/ash.2023.392

**Published:** 2023-09-29

**Authors:** Erin Hitchingham, Ashley Gambrell, Raquel Villegas, Daniel Muleta

## Abstract

**Background:** Clinical antibiotic susceptibility testing (AST) interpretations based on minimum inhibitory concentrations (MIC) breakpoints are important for both clinical decision making and some reportable condition criteria. Standardization of MIC breakpoints across clinical laboratories is lacking; AST instruments are often validated for outdated Clinical and Laboratory Standards Institute (CLSI) MIC breakpoint guidelines. In this study, we analyzed the agreement between the reported clinical laboratory AST interpretations and the guideline CLSI interpretation. **Methods:** Clinical laboratory AST data collected from the Multisite Gram-Negative Surveillance Initiative (MuGSI) carbapenem-resistant Enterobacterales (CRE) surveillance program in Tennessee between 2019 and 2021 were utilized. MIC values from the clinical instrument were used to calculate CLSI standard interpretations following the 2019–2021 CLSI M100 guidelines. Agreement between the clinical laboratory and CLSI interpretations of the reported MIC values were measured using a weighted Cohen κ calculated in SAS version 9.4 software. Total matches were isolates with identical CLSI and clinical laboratory interpretations. **Results:** In total, 14 antibiotics were assessed. Of those, 9 antibiotics had at least moderate agreement (κ > 0.41) between interpretations. Agreement between the clinical laboratory and the CLSI interpretations were near perfect (κ > 0.81) for 3 antibiotics. Agreement between the clinical laboratory and the CLSI interpretations were poor for cefazolin (0.06) and ertapenem (0.14). Cefotaxime (−0.07) was the only antibiotic that suggested no agreement. **Conclusions:** Of the antibiotics included in the analysis, 36% had less than moderate agreement between clinical laboratory and CLSI AST interpretations. Given the increases in antimicrobial resistance globally and the emphasis placed on antibiotic stewardship, standardization across clinical AST panels should be prioritized. Inconsistencies have the potential to contribute to inappropriate antibiotic use in addition to under- or overidentification of reportable conditions, including CRE.

**Figure:**
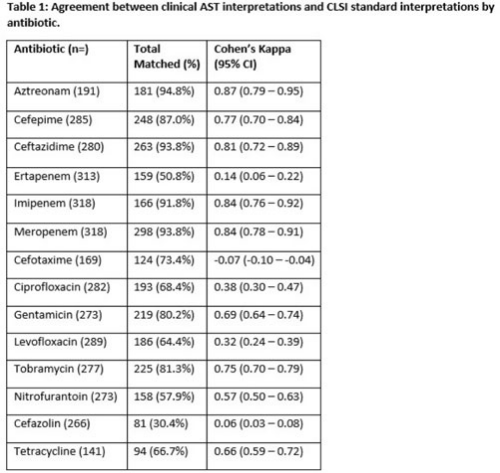

**Disclosures:** None

